# Comparative evaluation of flat-panel volume CT protocols for high-resolution visualization of middle ear anatomy in human skull specimens

**DOI:** 10.1038/s41598-025-33592-5

**Published:** 2026-01-05

**Authors:** Franz-Tassilo Müller-Graff, Jan von Düring, Bjoern Spahn, Stefan Weick, Anne Richter, Jan-Peter Grunz, Tilmann Neun, Stephan Hackenberg, Kristen Rak

**Affiliations:** 1 Department of Oto-Rhino-Laryngology, Head and Neck Surgery and the Comprehensive Hearing Center, University Hospital Wuerzburg, Josef-Schneider-Strasse 11, 97080 Wuerzburg, Germany; 2 Department of Radiotherapy and Radiation Oncology, University Hospital Wuerzburg, Wuerzburg, Germany; 3 Department of Diagnostic and Interventional Radiology, University Hospital Wuerzburg, Wuerzburg, Germany; 4 Institute for Diagnostic and Interventional Neuroradiology, University Hospital Wuerzburg, Wuerzburg, Germany

**Keywords:** Imaging protocols, Cumulative score, Reference structures, Gray-scale profile, Phantom dosimetry, Dose quantification, Ionization chamber, Secondary reconstruction of flat-panel volume CT (fpVCT_SECO_), Anatomy, Medical research

## Abstract

This study evaluates the imaging quality and radiation dose of six flat-panel volume CT protocols with varying technical parameters, including examination time (“Artis Icono” angiography system (Siemens Healthcare AG, Erlangen, Germany): 14s DCT HEAD MICRO CARE (14s I), 14s DCT HEAD MICRO CARE 4 (14s II), 8s DCT HEAD 70kV CARE 1 (8s I), 8s DCT HEAD 70kV CARE 3 (8s II), 4s DCT HEAD CARE FB (4s I), 4s DCT HEAD CARE FB 2 (4s II)) for middle ear visualization in human skull specimens. A scoring system assessed the delineation of 28 anatomical structures, narrowed to eight for detailed evaluation. Protocols 14s I and 8s I achieved the highest image quality, exceeding the clinical threshold. 8s I protocol demonstrated an optimal balance between image quality and radiation safety, adhering to the ALADA principle (“as low as diagnostically acceptable”). While 14s I offered superior detail, it involved higher radiation exposure without substantial further image improvement, indicating a saturation effect. Shorter protocols, such as 4s I, offered radiation-sparing advantages but did not consistently meet clinical image quality standards. Protocol 8s I is recommended as the standard for middle ear imaging, with 4s I as a potential alternative for cases requiring minimized radiation exposure and reduced motion artifact susceptibility.

**Supplementary Information:**

The online version contains supplementary material available at 10.1038/s41598-025-33592-5.

## Introduction

Modern imaging techniques such as digital volume tomography (DVT) and computed tomography (CT) play a crucial role in the diagnosis of middle ear pathologies^1–4^. Unlike multi-slice CT (MSCT), DVT systems employ a conical beam geometry, commonly referred to as Cone Beam CT (CBCT)^5,6^. The imaging capabilities of CBCT can be enhanced by incorporating flat-panel detectors, leading to significant improvements in imaging quality^7^. This combination is termed “flat-panel volume CT” (fpVCT)^8–10^. Secondary reconstructions from fpVCT (fpVCT_SECO_) have been shown to further enhance resolution and provide more accurate measurements, particularly in the context of cochlear implantation^11–13^.

However, a wide range of parameters such as voltage, amperage, and scan time in fpVCT can significantly affect both image quality and radiation dose. Higher voltage levels improve image contrast but also increase radiation exposure^14,15^, while longer scan times may result in greater motion artifacts. Pre-programmed fpVCT protocols define these key parameters, ultimately influencing image quality. Despite this, there is a limited body of research comparing the advantages and disadvantages of different fpVCT protocols for various clinical scenarios^16–19^.

Previous studies have compared fpVCT to MSCT for middle ear imaging, though caution is required when generalizing these results to clinical practice. Many studies have been conducted on temporal bone specimens^18,20–26^ or structural phantoms^27,28^, which do not fully replicate the real-world conditions of skull specimens^8,19,24^ or actual patient examinations^5,25,29–32^. Skull specimens, in particular, more closely simulate clinical scenarios by absorbing more low-energy photons, making them a more reliable model for imaging.

General comparisons of image quality between fpVCT and MSCT in middle ear imaging are challenging due to the vast array of manufacturers, models, and protocols. Nevertheless, the majority of studies suggest fpVCT as a preferred method for middle ear imaging^18,19,30^, although the evidence remains less conclusive compared to studies focused on inner ear imaging^11,13,17,30,33^.

Research on the selection of fpVCT protocols for middle ear imaging is scarce. Eisenhut et al. compared various protocols, recommending a 14-s protocol for high-resolution imaging, a 9-s protocol for routine postoperative assessments, and the 14-s protocol for specialized cases^18^. Reimann et al. found high-quality images from both 20-s and 10-s protocols, comparable to MSCT^19^. However, studies on shorter protocols (4 to 14 s) in the context of middle ear imaging remain limited. Additionally, prior research did not utilize whole-head specimens^18^, nor did they incorporate direct radiation dosimetry, which is essential for the final decision-making process regarding clinical application. Furthermore, none of these studies involved secondary reconstructions, which are known to enhance image quality.

The aim of this study is to evaluate the performance of various fpVCT protocols for middle ear imaging using human skull specimens. The study seeks to investigate the influence of these protocols on image quality and to determine the associated radiation doses. Furthermore, the quantified radiation doses will be compared against the measured image quality to establish the optimal balance for clinical use.

## Results

### Preparation of various imaging protocols of fpVCT

**Fig. 1:**
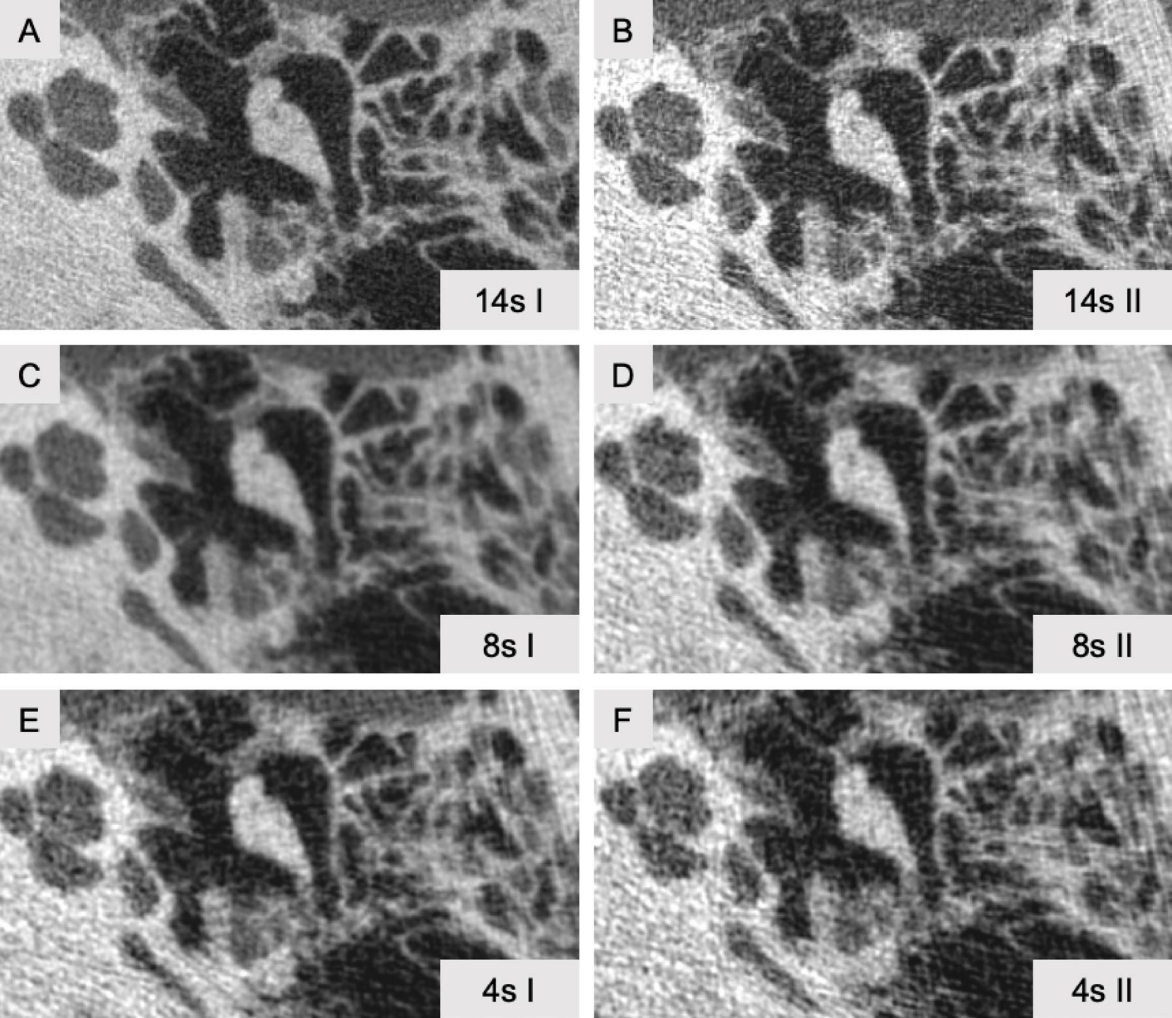
Visualization of the six different fpVCT protocols examined. The protocols are illustrated with examination times of 14 s (**A**) and (**B**), 8 s (**C**) and (**D**), and 4 s (**E**) and (**F**). Protocols with higher voltage are designated as “I”, while those with lower voltage are designated as “II”. Specifically: 14s I = 14s DCT HEAD MICRO CARE, 14s II = 14S DCT HEAD MICRO CARE 4, 8s I = 8S DCT HEAD 70kV CARE 1, 8s II = 8S DCT HEAD 70kV CARE 3, 4s I = 4S DCT HEAD CARE FB, 4s II = 4S DCT HEAD CARE FB 2, each with a slice thickness of 100µm. fpVCT = flat-panel volume CT. Window width = 4588 HU; window level = 844 HU.

The image quality varied depending on the different imaging protocols applied (Fig. 1). For example, the crura stapedis were clearly delineated in the images obtained with protocols 14s I and 8s I. However, with protocol 8s II, the continuity, form, and shape of the bones could no longer be definitively established. Protocol 4s II provided no reliable information regarding the stapes morphology.

### Selection of the most meaningful structures

**Fig. 2:**
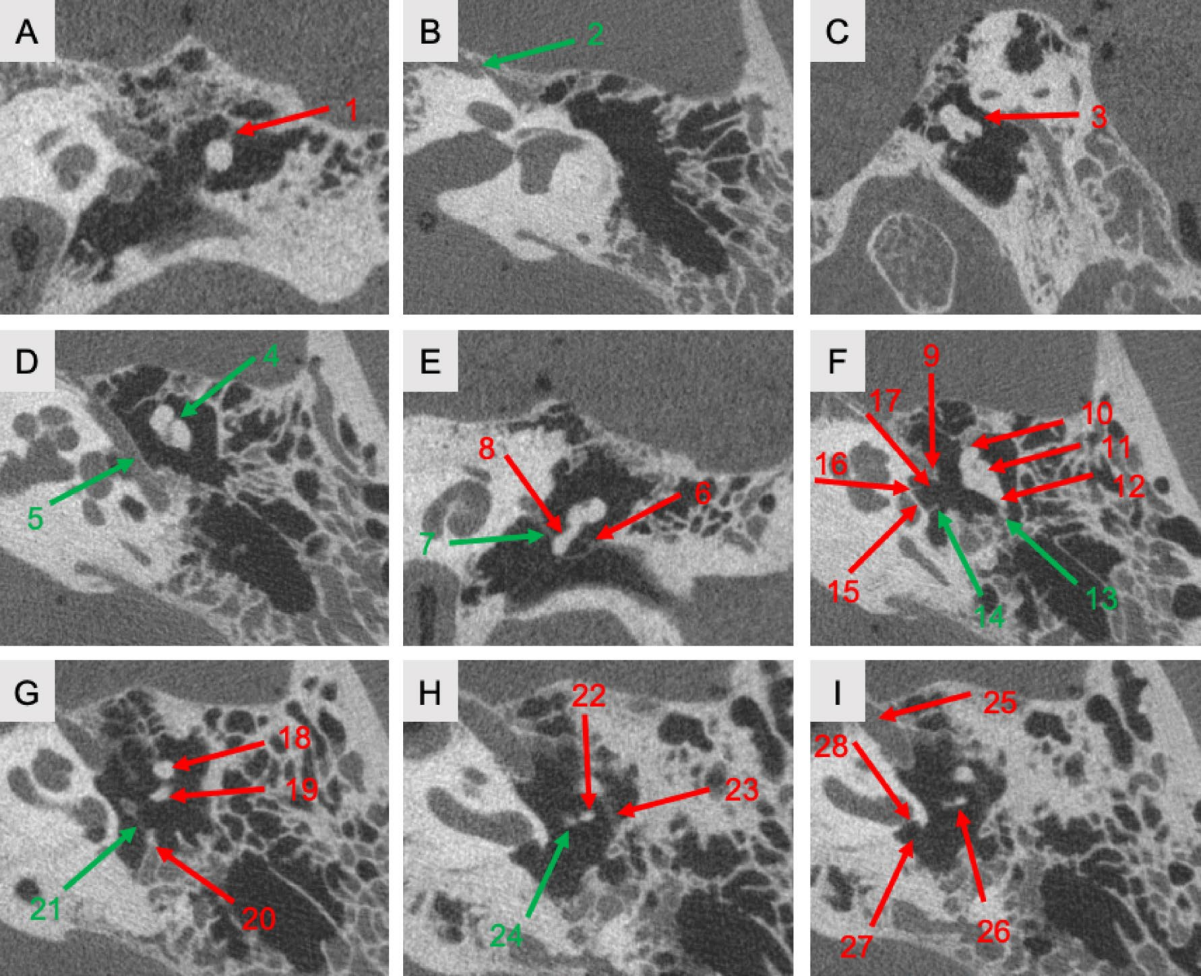
28 examined anatomical structures in the middle ear area. The 28 anatomical structures in the middle ear area, which were evaluated for their delineation by the score, are labeled and numbered with arrows. Green arrows represent the most suitable structures. Soft tissue structures (e.g., chorda tympani, ligaments) were not evaluated based on their soft-tissue contrast, but indirectly via their anatomical course, bony canal or osseous anchoring sites. 1 = Ligamentum mallei superius, 2 = Canalis nervi petrosi majoris, 3 = Ligamentum incudis superius, 4 = Articulatio incudomallearis, 5 = Prominentia canalis nervi facialis, 6 = Ligamentum mallei laterale, 7 = Musculus tensor tympani (tympanal), 8 = Manubrium mallei, 9 = Processus cochleariformis, 10 = Ligamentum mallei anterius, 11 = Corpus incudis, 12 = Crus breve incudis, 13 = Ligamentum incudis posterius, 14 = Crus posterius stapedis, 15 = Basis stapedis, 16 = Crus anterius stapedis, 17 = Fenestra ovalis, 18 = Caput mallei, 19 = Crus longum incudis, 20 = Eminentia pyramidalis, 21 = Musculus stapedius, 22 = Processus lenticularis, 23 = Chorda tympani, 24 = Caput stapedis, 25 = Musculus tensor tympani in the Semincanalis musculi tensoris tympani, 26 = Articulatio incudostapedialis, 27 = Fenestra rotunda, 28 = Membrana tympanica secundaria. Flat panel volume CT. Protocol: 14s I; Slice thickness: 100µm. Green arrows represent the meaningful selected structures. Window width = 4588 HU; window level = 844 HU.

In the first analysis, the suitability of 28 anatomical structures of the middle ear for further investigation of image quality differences was evaluated (all 28 structures are visualized in Fig. 2). Some structures scored higher than others, particularly larger bony structures with clear contrast to the surrounding air, such as the fenestra rotunda (3.00) and the corpus incudis (3.00). Smaller, less dense structures, such as joints, ligaments, muscles, and nerves, scored lower, for example, the articulatio incudostapedialis (1.38), ligamentum mallei laterale (0.38), musculus stapedius (0.88), and chorda tympani (0.88). The standard deviation (SD) ranged between 0 and 0.95, varying depending on the demarcation of the structures. Some structures exhibited a low SD, such as the crus breve incudes (0.25), while others showed greater variability, such as the ligamentum incudis posterius (0.89). Similarly, the maximum-minimum difference (MMD) varied between 0 and 3, such as the corpus incudis (0) and the processus lenticularis (3.00). All examined structures with mean score, SD and MMD as well as visualization of the descriptive statistics can be found in suppl. Table 1 and suppl. Figure 1.

Only the most suitable structures were selected for further analysis. Priority was given to structures with (I) a high SD and (II) a large MMD across different protocols and slice thicknesses. This was complemented by (III) low to average scores averaged across modalities, which reflected the general delineation of the structures. Additionally, clinically relevant structures with varied radiographic densities, including joints, muscles, bones, nerves, and ligaments, were considered. The final selection of 8 structures was made as the optimal compromise based on these criteria (These are visualized with green arrows in Fig. 2):

(I) prominentia canalis nervi facialis (mean score: 1.31; SD: 0.95; MMD: 3.00)
(II) canalis nervi petrosi majoris (mean score: 1.50; SD: 0.89; MMD: 3.00)
(III) ligamentum incudis posterius (mean score: 1.50; SD: 0.89; MMD: 3.00)
(IV) crus posterius stapedis (mean score: 1.13; SD: 0.72; MMD: 2.00)
(V) musculus tensor tympani (mean score: 2.00; SD: 0.63; MMD: 2.00)
(VI) musculus stapedius (mean score: 0.88; SD: 0.72; MMD: 2.00)
(VII) articulatio incudomallearis (mean score: 1.56; SD: 0.73; MMD: 2.00)
(VIII) caput stapedis (mean score: 1.38; SD: 0.62; MMD: 2.00)

### Evaluation of the protocols

**Fig. 3:**
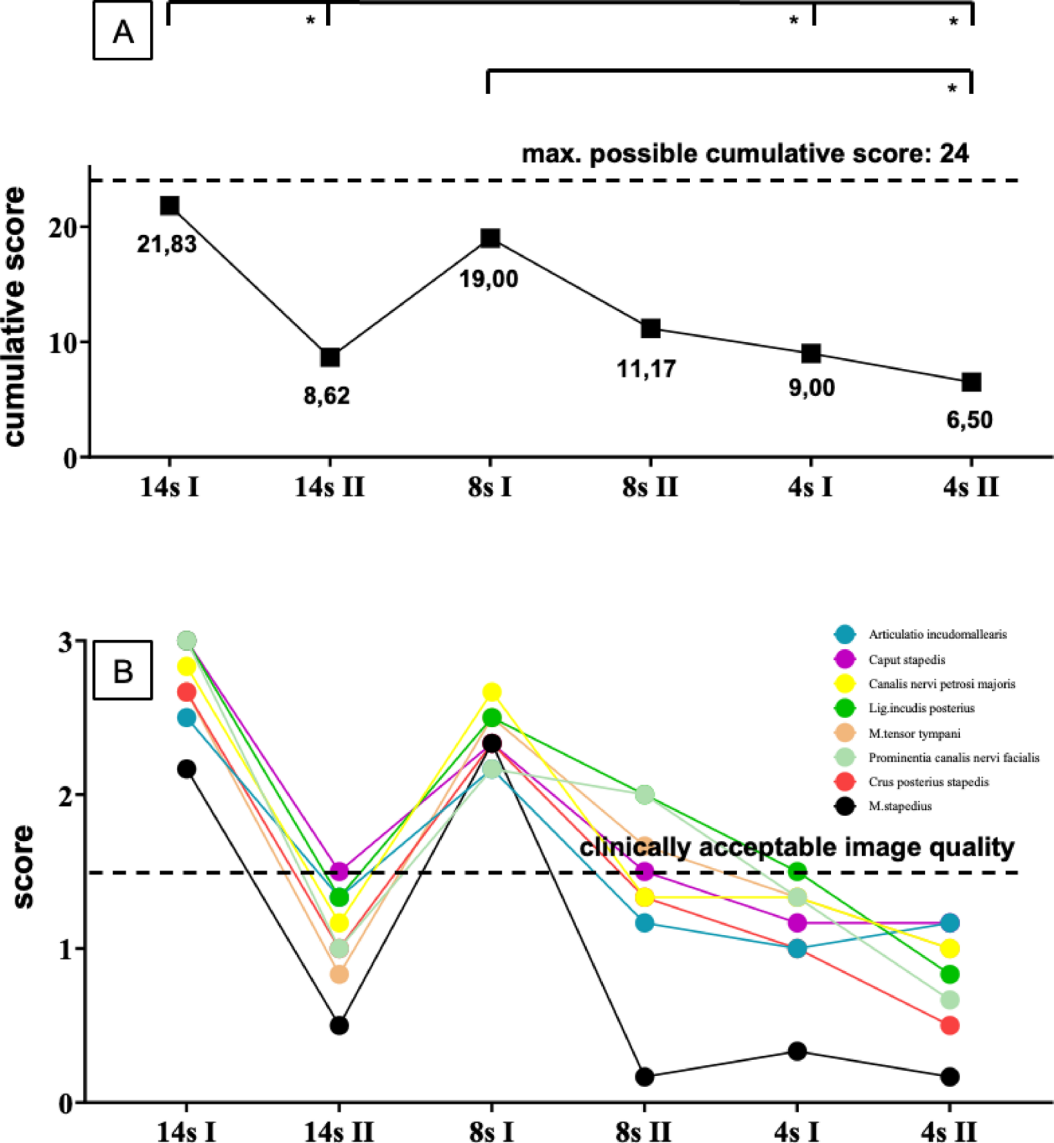
Evaluation of image quality across six fpVCT-protocols. (**A**) “Cumulative score” as a function of the protocol used. The cumulative score represents the sum of the scores (reference range: 0–3) for eight anatomical structures within each protocol (maximum achievable score: 24). Significant differences are indicated (*p ≤ 0.05); Friedman test with pairwise comparison. (**B**) Illustration of the eight individual structures contributing to the cumulative score. The color-coded legend indicates the corresponding middle ear structures. The dashed line represents the threshold for clinically acceptable image quality. Raters: n = 3. fpVCT = flat-panel volume CT.

In the second analysis, the highest cumulative score (8 structures rated from 0 to 3) was observed in protocol 14s I (21.83; reference value: 0–24), while the lowest score was found in protocol 4s II (6.50; reference value: 0–24). Significant differences in image quality were identified between the protocols 8s I and 4s II (*p* ≤ 0.008),14s I and 14s II (*p* ≤ 0.006), 14s I and 4s I (*p* ≤ 0.03) and 14s I and 4s II (*p* ≤ 0.001). The results of this evaluation are illustrated in Fig. 3A.

Additionally, the demarcation of the eight anatomical structures under investigation was analyzed. Some structures exhibited better delineation across all protocols, such as the canalis nervi petrosi majoris, while others, like the musculus stapedius were more challenging to assess. In the scoring system, a threshold between clinically acceptable (2–3) and unacceptable image quality (0–1) was established. This threshold represented the limit above which an anatomical structure could be reliably identified, with its morphology fully recorded and accurately delineated. Since the transition between acceptable and unacceptable image quality occurred between scores of 1 and 2, this limit was set at 1.5. Only the 14s I and 8s I protocols provided reliable delineation of all eight structures according to the defined threshold. The image properties of protocols 14s II, 8s II, and 4s I enabled reliable demarcation of some structures, whereas all structures fell below this threshold in protocol 4s II. The delineation of each individual structure, contributing to the “cumulative score” (Fig. 3A), is visualized according to the protocols in Fig. 3B.

### Dose measurement using phantom model and ionization chamber

The highest radiation dose (CT dose index (CTDI_w_)) was recorded for protocol 14s I (24.82 mGy), while the lowest dose was observed for protocol 4s II (3.47 mGy). For the same examination time, protocol 14s I delivered a significantly higher dose compared to protocol 14s II (5.74 mGy). Notably, protocols 14s II, 8s II, 4s I, and 4s II exhibited similar radiation doses, whereas the 14s I and 8s I protocols (15.04 mGy) were more radiation-intensive. The measured charge values at the various measurement points within the phantom skull, along with the corresponding absorbed radiation doses and calculated total CTDI_w_ doses for each protocol, are presented in Table 1.

**Table 1:** Results of the test setup for determining the charge and total radiation dose exposure from the six evaluated fpVCT protocols.

Charge [pC]					
	Measuring point 1	Measuring point 2	Measuring point 3	Measuring point 4	
14 s I	47.00	55.90	99.90	121.00	
14 s II	12.00	14.78	22.50	25.84	
8 s I	51.40	33.12	52.17	63.73	
8 s II	24.40	18.57	23.50	30.40	
4 s I	21.50	11.57	21.70	27.70	
4 s II	12.10	7.42	14.25	11.31	

Absorbed dose [mGy]					
	Measuring point 1	Measuring point 2	Measuring point 3	Measuring point 4	CTDI_W_
14 s I	14.05	16.71	29.86	36.17	24.82
14 s II	3.59	4.42	6.72	7.72	5.74
8 s I	15.36	9.90	15.59	19.05	15.04
8 s II	7.29	5.55	7.02	9.09	7.21
4 s I	6.43	3.46	6.49	8.28	6.20
4 s II	3.62	2.22	4.26	3.38	3.47

### Relationships between image quality, examination time, and radiation dose

**Fig. 4:**
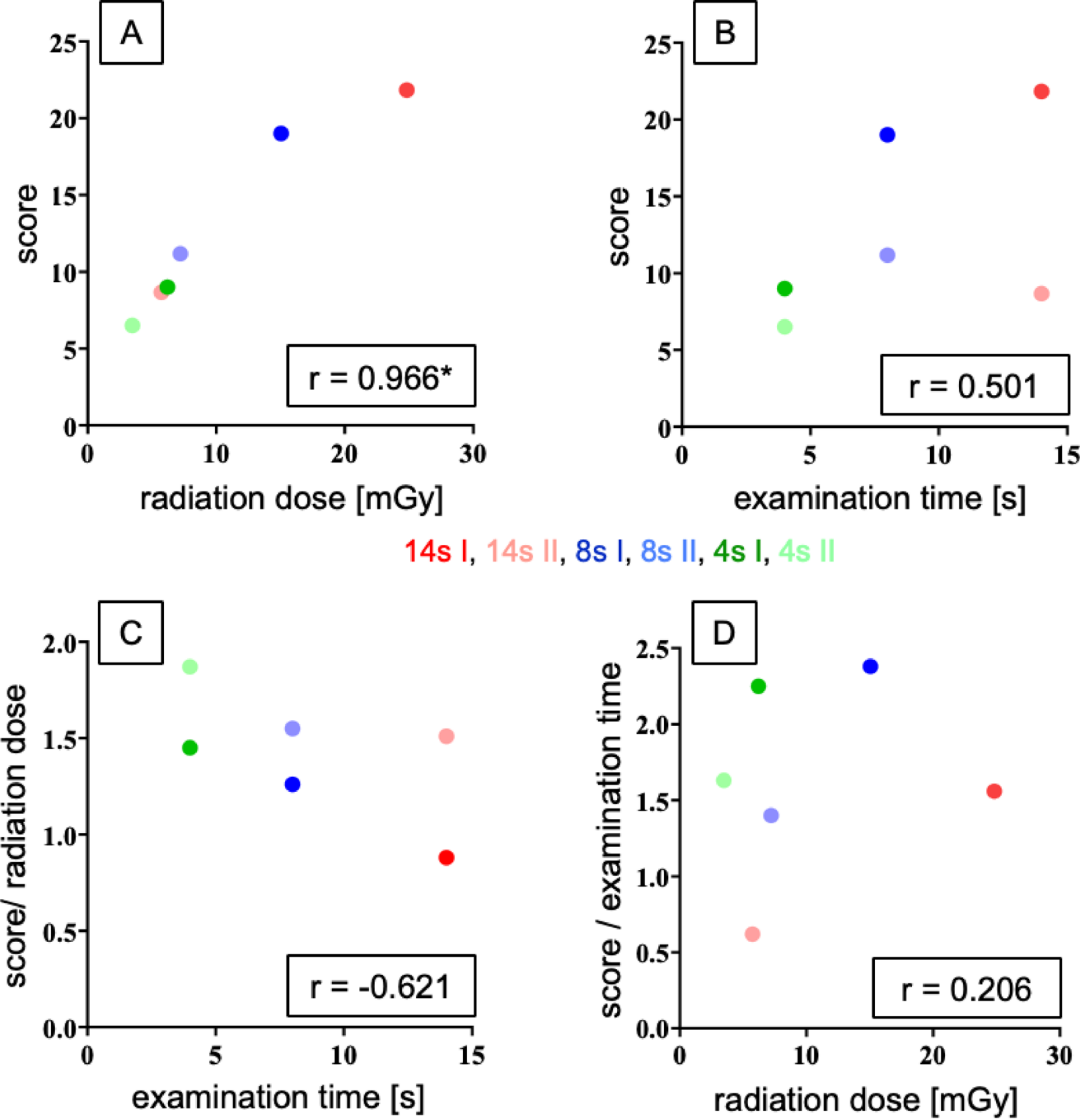
Relationships between cumulative score, radiation dose, and examination time across the six evaluated fpVCT protocols. Comparison of the cumulative score with radiation dose [mGy] (**A**) and examination time [s] (**B**). The ratio of cumulative score to radiation dose is related to examination time (**C**), and the ratio of score to examination time is related to radiation dose (**D**). Bivariate correlations were determined using Pearson’s method. fpVCT = flat-panel volume CT.

The variables (I) image quality, presented as the cumulative score (reference value 0–24), (II) examination time (4s, 8s and 14s) and (III) radiation dose were compared across the six protocols examined, and various ratios were calculated (Fig. 4).

A nearly linear and significant correlation between cumulative score and radiation dose was found (r = 0.966; *p = 0.02), with protocols delivering higher doses generally yielding higher scores. However, a saturation effect on image quality was observed in the dose range between the 8s I and 14s I protocols (Fig. 4A). No linear correlation between cumulative score and examination time was found, although longer examination times tended to produce higher cumulative scores (r = 0.501; *p* = 0.311) (Fig. 4B). Lower-dose protocols showed a better cumulative score-dose ratio for the same examination time compared to higher-dose protocols. This ratio decreased with increasing dose, particularly for protocols 4s I, 8s I, and 14s I. Protocol 8s II was an exception, demonstrating a high score-dose ratio despite a low dose. Increasing the examination time was generally associated with a decrease in the score-to-dose ratio (r = − 0.621; *p* = 0.188) (Fig. 4C). Protocols with high cumulative scores and low examination times, particularly protocol 8s I, were located at the top of the y-axis. In contrast, protocol 14s II exhibited a low cumulative score-to-examination time ratio. The quotient of cumulative score to examination time showed a weak positive correlation with the radiation dose (r = 0.206; *p* = 0.695) (Fig. 4D).

### Gray-scale profile analysis

**Fig. 5:**
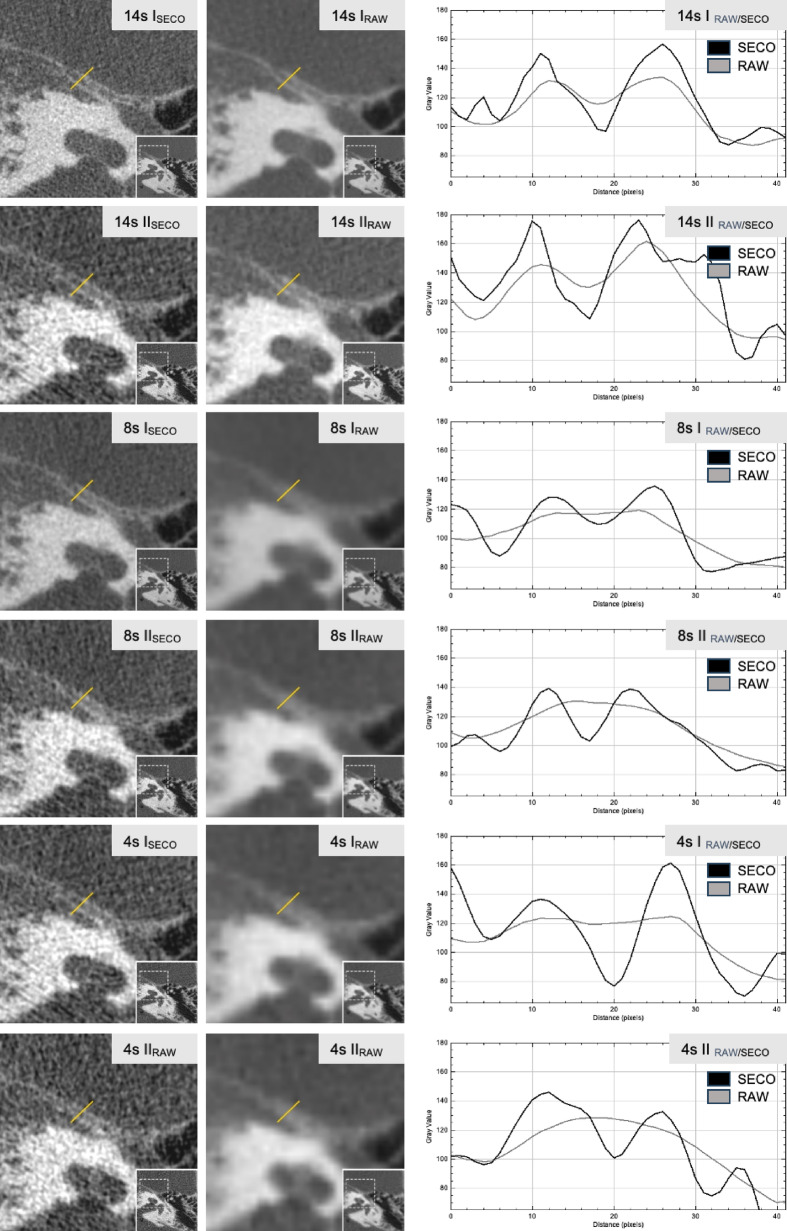
Visualization of the image quality of the six fpVCT-protocols, represented by gray-scale profiles. On the left, the canalis nervi petrosi majoris is depicted in various fpVCT protocols using image files from fpVCT_SECO_ with a slice thickness of 100 µm, as well as for illustrative purposes, in fpVCT image files without secondary reconstruction (fpVCT_RAW_). Overview images of the temporal bone are shown in the bottom right corner of these images. On the right, gray values were recorded in the corresponding areas (yellow markers) of the fpVCT and graphed for comparison between the two imaging modalities. Gray: fpVCT_RAW_, black: fpVCT_SECO_; fpVCT_RAW_ = flat panel volume CT without secondary reconstruction; fpVCT_SECO_ = secondary reconstruction of the flat panel volume CT with a slice thickness of 100 µm. Window width = 4588 HU; Window level = 844 HU.

Gray-scale profiles were utilized to visually illustrate differences in image quality across the six fpVCT protocols, comparing image files of fpVCT_SECO_ with a slice thickness of 100 µm and, for better illustration, fpVCT image files without secondary reconstruction (fpVCT_RAW_). The bony borders of the canalis nervi petrosi majoris were selected as an example. The fpVCT_SECO_ protocol provided a more precise visualization of the anatomy compared to fpVCT_RAW_. While in some protocols the peaks in the gray-scale profiles merged, resulting in unclear anatomical structures, other protocols exhibited distinct elevations of the bony structures (Fig. 5).

## Discussion

### Selection of the most meaningful structures

A preliminary selection of 28 middle ear structures was narrowed down to eight, as these were deemed most suitable for differentiating between the various fpVCT protocols (Fig. 2, suppl. Figure 1 and suppl. Table 1). Previous studies have assessed temporal bone and middle ear structures to evaluate imaging protocols. However, a systematic reduction of structures based on methodological criteria has not been documented^17–19,29,31,34,35^. The structures analyzed in this study align with those selected in other studies^5,18,24,29,34^, indicating that similar considerations regarding the advantages and limitations of different anatomical features in imaging were likely applied.

The selection process in this study employed a scoring system, prioritizing structures with lower scores due to their potential for improved differentiation with higher image quality. Additional factors, including the standard deviation of scores, maximum-minimum differences, clinical relevance, and variations in density values, were considered. Despite occasional contradictions, multiple criteria were methodically weighed, leading to the selection of eight structures. This structured approach enhances the study’s significance and provides reference structures for future imaging studies.

### 14s I and 8s I as protocols of clinically acceptable image quality

Data analysis revealed that the 14s I protocol achieved the highest image quality, as indicated by cumulative scores, followed by the 8s I protocol. These were the only protocols that met clinically acceptable image quality standards for all eight examined structures. In contrast, other protocols failed to meet this standard: 14s II, 8s II, and 4s I protocols exhibited suboptimal image quality for certain structures, while the 4s II protocol did not meet the minimum standard for any examined structure (Fig. 3). These findings are further corroborated by gray-scale profiles (Fig. 5).

Few studies have compared image characteristics and radiation doses across different fpVCT protocols for middle ear imaging. Reimann et al. and Eisenhut et al. demonstrated that newer fpVCT devices with shorter examination times (e.g., 14-s protocols) tend to produce better image quality than older systems with longer scan durations (e.g., 20-s protocols)^18,19^. This improvement was attributed to higher frame rates and faster C-arm rotations in newer devices. While the authors also evaluated protocols with examination times of 9 and 7 s, these were unable to match the image quality achieved with the 14-s protocol^18^. Consistent with these findings, the present study confirmed that 14-s protocols deliver superior image quality compared to shorter protocols of 8 s and, for the first time, evaluated and demonstrated inferior results for 4-s protocols. These data suggest that longer examination times, such as the 20-s protocols used in older systems, are no longer necessary.

Beyond examination time, technical parameters such as voltage and amperage substantially influence image quality in fpVCT. Notably, the 14s I protocol achieved a significantly higher cumulative score than the 14s II protocol, despite identical examination times. This discrepancy can be attributed to voltage differences (90 kV for 14s I versus 71 kV for 14s II), while amperage values were 356 mA for protocol 14s I and 402 mA for protocol 14s II. The higher voltage in the 14s I protocol contributed to better image quality but also increased radiation exposure. This is due to the fact that the amperage is proportional to the radiation dose and the voltage is proportional to the square of the radiation dose^15^. This example underscores the complex interplay between technical parameters, image quality, and radiation dose.

### Radiation dose analysis of fpVCT imaging protocols

**Fig. 6:**
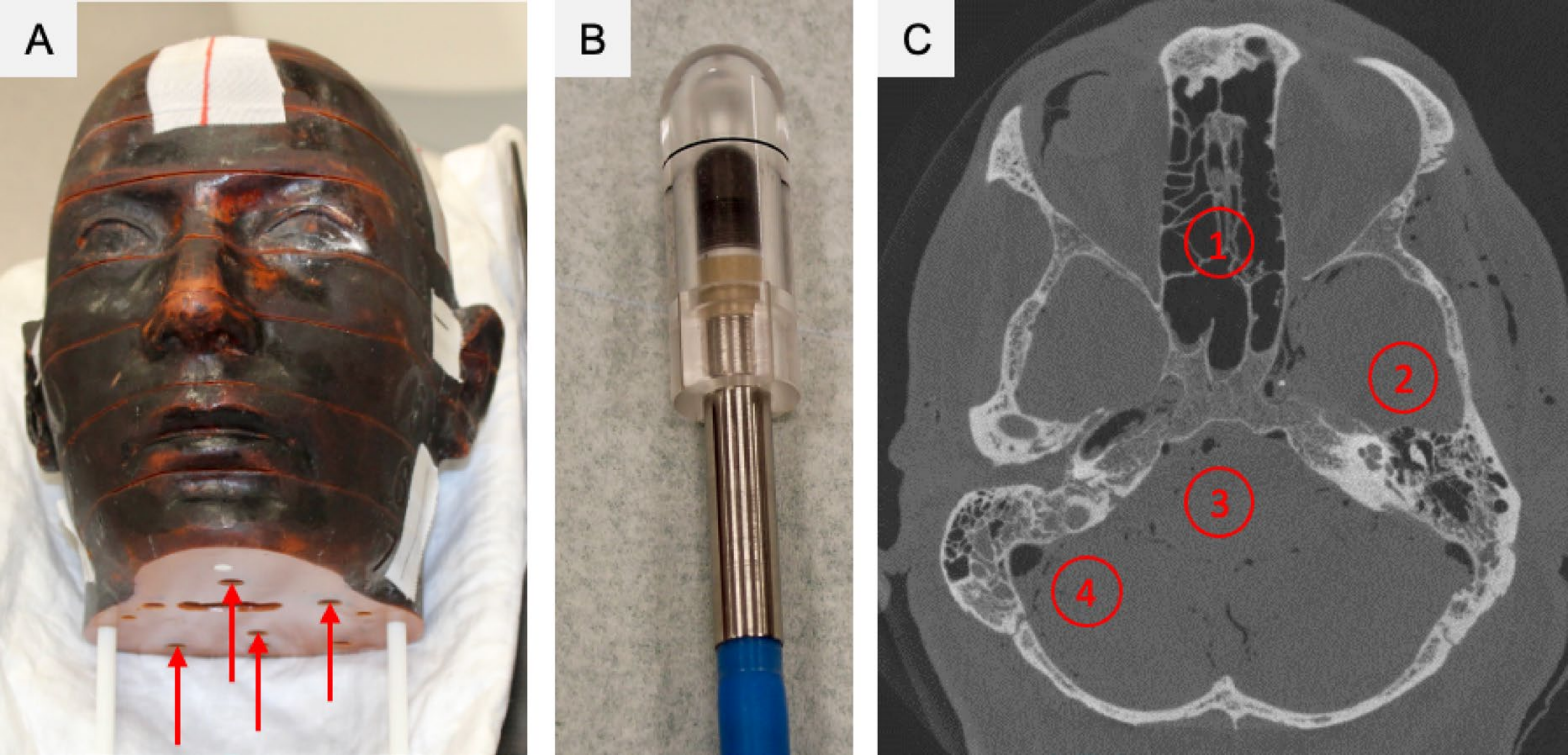
*Phantom model and ionization chamber used for measuring the applied imaging radiation dose.* (**A**) The anthropomorphic phantom model RANDO (Alderson Research Laboratories Inc., Stamford, CT, USA). (**B**) The ionization chamber (Semiflex Ionization Chamber 31010, PTW, Freiburg, Germany(**C**) Exemplary overview image of a skull specimen acquired using the 8s I protocol of fpVCT, with red circles indicating the measuring points for the CTDI_w_ radiation dose. Measuring points 1 and 3 correspond to the central measuring points (CTDI_C_), while points 2 and 4 represent the peripheral measuring points (CTDI_P_). CTDI = CT dose index. fpVCT = flat panel volume CT.

Radiation dose measurements were conducted using a test setup (Fig. 6* and *Table 1). The 14s I (24.82 mGy) and 8s I (15.04 mGy) protocols not only demonstrated the highest image quality but also exhibited significantly higher radiation doses compared to other protocols (Table 1).

The literature on radiation dose comparisons between fpVCT and the more established MSCT is varied and intensively discussed. Studies often use different measurement parameters such as CT dose index (CTDI)^17,18,27,28,34,36,37^, dose length product (DLP)^30^, dose area product (DAP)^29,38^,or effective dose^17,36^, with CTDI being the most commonly employed metric. Comparisons are complicated by the variability of these measurement methods. For instance, Knörgen et al. reported lower doses for fpVCT (8.0 mGy) compared to MSCT (29.9 mGy)^27^, while Eisenhut et al. found that fpVCT doses could exceed those of MSCT (27.5mGy), with values as high as 166 mGy for the 14s protocol, but also with shorter protocols, such as the 7s fpVCT protocol (37.5 mGy)^18^. Kyriakou et al. observed minimal differences between fpVCT and MSCT systems, with radiation exposures ranging from 2.3 mGy to 3.1 mGy^28^. The radiation doses observed in the present study (3.47 mGy to 24.82 mGy) fall within the lower range of this spectrum (Table 1).

Even this very short summary of several studies demonstrates the variability, which underscores the influence of manufacturers, devices, and protocols on radiation doses. Moreover, differences in measurement methods and test setups complicate comparisons. The present study’s test arrangement is considered more reliable than equipment-generated dose reports. However, variations in materials and experimental setups remain, making standardization and classification of fpVCT protocols challenging.

### Consideration of key image parameters

Comparative analysis revealed a strong correlation between cumulative image quality scores and radiation dose, but a less distinct relationship between image quality and examination time. Notably, the quotient of image quality to radiation dose exhibited a weak negative correlation with examination time, while no correlation was found between the quotient of image quality to examination time and radiation dose (Fig. 4).

Figure 4A indicates a saturation effect, where image quality no longer increases proportionally with radiation dose beyond the 8s I protocol. Thus, the 14s I protocol appears less favorable than the 8s I protocol, which remains within the linear improvement range. Figure 4B demonstrates a weak correlation between score and examination time, indicating that longer scan durations do not necessarily enhance image quality. Conversely, the 4-s protocols also failed to deliver improved image quality, likely due to the limited number of captured frames at a fixed frame rate of 68 frames per second. Technological advancements enabling higher frame rates could improve image quality and benefit patients who struggle to remain motionless.

Figure 4C highlights a more favorable dose-to-score ratio for protocols with lower image quality, indicating a saturation effect between these variables. Protocol 14s II exhibited a high examination time but a lower radiation intensity, yielding a favorable ratio. The 4s I and 4s II protocols emerged as potential options due to their balanced dose-to-score ratios and short examination times.

Considering the increased risk of motion artifacts with longer examination times, the ratio of cumulative score and examination time to radiation dose was also analyzed (Fig. 4D). Protocol 8s I demonstrated the most favorable profile, enabling high differentiation with a moderate examination time. Protocol 14s II, on the other hand, showed an unfavorable ratio. From a radiation safety perspective, protocol 14s I was less favorable than 8s I, and this in turn appears less favorable than 4s I.

### Clinical relevance and proposed use of imaging modalities

The study focused on assessing the image quality of different fpVCT-protocols (Fig. 1), while considering their associated radiation doses and examination time to provide clinical recommendations. The findings indicate that only protocols 14s I and 8s I allowed reliable delineation of all anatomical structures, with protocol 14s I offering the highest level of detail. However, the lack of proportional image quality improvement with increased radiation dose renders 14s I less desirable from a radiation safety standpoint.

In line with the ALADA principle (“as low as diagnostically acceptable”), protocol 8s I is preferable^34^, as it offers a balanced trade-off between image quality and examination time. This is particularly relevant since the study utilized anatomical specimens without motion artifacts. In clinical practice, longer protocols may be more susceptible to motion artifacts, potentially compromising image quality.

Protocol 4s I also has advantages, including a high image-quality-to-radiation-dose ratio and a remarkably efficient examination time. This makes it a compelling option from a radiation protection point of view and for cases involving motion artifacts. However, its lower image quality must be weighted carefully against these benefits in adherence to the ALADA principle^34^.

Given its superior image quality and moderate radiation exposure, protocol 8s I should be regarded as the standard protocol. Protocol 14s I delivers exceptional image detail but carries higher risks due to its longer examination time, increased radiation dose and susceptibility to motion artifacts. Thus, it is recommended only for specific cases when patients can remain motionless for the full 14-s duration.

While other protocols did not meet the minimum criteria for acceptable image quality, protocol 4s I may be considered in situations where minimizing radiation exposure and motion artefacts are priorities, such as in pediatric imaging. However, its image quality currently remains insufficient for general clinical recommendation.

A limitation of this study is the use of only two non-pathological whole-head cadaver specimens. Therefore, the transferability of the results to routine clinical practice, particularly in cases with pathological alterations, remains restricted.

## Conclusion

This study evaluated the image quality, radiation dose, and examination time of various fpVCT protocols, with a focus on determining the most clinically relevant options. Protocol 14s I demonstrated the highest image quality, but also carried a significantly higher radiation dose and longer examination time, making it less favorable from a radiation safety perspective. Protocol 8s I, which offers a balanced trade-off between image quality and examination time, is recommended as the standard protocol and represents the optimal choice for clinical practice, as it provides sufficient detail while adhering to the ALADA principle (“as low as diagnostically acceptable”). Protocol 4s I, despite its lower image quality, offers advantages in terms of radiation dose and examination efficiency, making it suitable for situations involving motion artifacts.

## Methods

### Skull preparations

Two formaldehyde-fixed whole-head human specimens were utilized for fpVCT imaging. The anatomical specimens were supplied by the Anatomical Institute of our university to the Department of Oto-Rhino-Laryngology, Head and Neck Surgery at the University Hospital Wuerzburg, Germany, for educational and research purposes. The local ethics committee approved this study (ID: 2020111704). The body donors had provided written informed consent during their lifetime to be included in experimental research; therefore, no additional written informed consent was required. During all examinations, ethical protocols were strictly followed in compliance with state and federal laws and the principles of the “WMA Declaration of Helsinki on Ethical Principles for Research Involving Human Subjects”, ensuring the proper acquisition and use of human specimens. Care was taken to ensure that the specimens exhibited no injuries in the temporal bone region and that the tympanic cavity was free of fixation fluid. Accordingly, only the left side of each skull specimen could be included in the study. Both specimens exhibited no malformations in the region of the temporal bone.

### Imaging with flat-panel volume CT

#### Acquisition of image files

The scans were obtained using the “Artis Icono” angiography system (Siemens Healthcare AG, Erlangen, Germany). A total of six different protocols with varying technical parameters were utilized (Table 2). In particular, the parameters (i) examination time, (ii) frame rate, (iii) voltage and (iv) amperage determine the characteristics of a protocol. The examination time is particularly critical as it influences the likelihood of motion artifacts. For this study, two protocols were selected for each of the three examination times (4, 8 and 14 s). The protocol with the higher voltage was labeled as “I” and the protocol with the lower voltage as “II”. These protocols were chosen to cover a sufficiently broad range of parameter variations. To simulate clinical conditions during fpVCT imaging, the aperture was adjusted to fit the respective temporal bone for each scan. Additionally, a foam wedge was placed under the samples to replicate a cervical spine tilt of 20 degrees.

**Table 2:** Technical parameters of the 6 fpVCT-protocols examined.

Protocol	14s I	14s II	8s I	8s II	4s I	4s II
Protocol name of the device manufacturer	14S DCT HEAD MICRO CARE	14S DCT HEAD MICRO CARE 4	8S DCT HEAD 70kV CARE 1	8S DCT HEAD 70 kV CARE 3	4S DCT HEAD CARE FB	4S DCT HEAD 70 kV CARE 2
Voltage [kV]	90	79	85	71	76	71
Amperage [mA]	356	402	391	383	427	398
Examination time [s]	14	14	8	8	4	4
Zoom [cm] (scan field of view)	25	25	49	49	49	49
Number of images	496	496	496	496	248	248
Pulse width [ms]	8	8	4	4	4	4
Frame rate [B/s]	40	40	68	68	68	68

#### Creation of secondary reconstructions of the fpVCT

Secondary reconstructions (fpVCT_SECO_) were created from the raw fpVCT image files (fpVCT_RAW_), resulting in a reduction of the volume of interest (VOI) while maintaining the same matrix, which increases the resolution^11,33,39^. According to the findings of Pearl et al. the following settings were used for all protocols: 512 × 512 section matrix; HU kernel types; sharp image characteristics; slice thickness = 100 μm^39^. Isotropic voxel size: 0.10 × 0.10 × 0.10 mm. The reconstructed imaging volume extended 60 mm in the cranio-caudal direction. All datasets were reconstructed using standard filtered back projection.

Furthermore, fpVCT_SECO_ of the protocols with a slice thickness of 400 µm were generated for comparison purposes. However, this was only feasible for the 8s I, 8s II, 4s I and 4s II protocols, but not with the 14s I and 14s II protocols, which was due to the smaller zoom of 25 cm of these protocols (Table 2), as the long, high-resolution fpVCT modes are technically limited to a smaller scan field of view due to the higher projection density and reconstruction matrix requirements.

#### Dose measurement using a phantom model and ionization chamber

An experimental setup was developed using an anthropomorphic phantom model equipped with an ionization chamber to accurately measure the applied imaging dose. The skull of the RANDO phantom model (Alderson Research Laboratories Inc., Stamford, CT, USA) was employed for this setup (Fig. 6A). This model consists of tissue-equivalent plastic, allowing for the positioning of the ionization chamber at multiple points in the axial plane of the temporal bone to measure dose values (Fig. 6C). The ionization chamber (Semiflex Ionization Chamber 31010, PTW, Freiburg, Germany) (Fig. 6B) was connected to a dosimeter (Unidos S3, PTW, Freiburg, Germany) for charge readings in picocoulombs [pC]. Absorbed dose values (in mGy) for each examination protocol were determined at four measurement points in the phantom using the following equation:

$$Dose = C * N * k\_p * 10^{ - 9}$$

C = charge [pC];

N = calibration factor of the ionization chamber of 289900000 [Gy/C];

K_p = daily correction factor of air pressure and temperature (at the time of measurement of 1.031)^40^.

From these absorbed dose values, the CT dose index (CTDI) was subsequently calculated, as it is the most frequently used physical metric in literature for assessing radiation exposure from fpVCT^17,18,27,28,34,36,37^. Although CTDI is typically measured in standardized cylindrical phantoms, we applied the same principle to the RANDO head phantom. Since all fpVCT protocols were assessed under identical conditions, comparability of the results is preserved. Both the size of the phantom and the positioning of the measurement points affect the CTDI. To account for these factors, the position-weighted CTDI_W_^41^ was calculated using the following equation:

$$CTDI_{W} = \frac{1}{3}CTDI_{C} + \frac{2}{3}CTDI_{P}$$

In this measurement setup, points 1 and 3 represented central measuring points (CTDI_C_), while points 2 and 4 represented peripheral measuring points (CTDI_P_).

### Subjective examination using a scoring system

To evaluate the suitability of various middle ear structures for distinguishing image quality differences between imaging modalities, anatomical references from textbooks^42–44^ and structures previously used in related studies^5,8,18–20,34,45–47^ were selected. Special consideration was given to diversifying the structures based on their morphology, function, and radiological density. 28 key structures were categorized into the following subgroups: (1) ligaments of the ossicles, (2) muscles of the ossicles, (3) joints of the ossicles, (4) ossicles, (5), and (6) bony structures. Because soft tissues are not directly visualized in high-contrast CT, they were evaluated based on their course and corresponding bony boundaries. These structures are visualized and labeled in Fig. 2.

To quantify structural differences, a four-level scoring system was adopted, as recommended by similar methodological studies^18,30^. This approach established a symmetrical scale, delineating the threshold between clinically acceptable and unacceptable image quality. The score was defined as follows: 0: Not definable or unreliable; 1: Poor definability; 2: Good definability; 3: Very good definability. Each score was strictly related to the specific structure under evaluation, excluding the overall image impression or unrelated structures.

Initially, all 28 structures were evaluated as a screening step to highlight differences in image quality across modalities and to identify structures that are robustly visualized under varying imaging conditions. The first scoring round was performed by one examiner (medical doctor trained in otologic imaging, under supervision of an experienced neuroradiologist), who assessed mean score, standard deviation (SD), and maximum-minimum differences (MMD) for slice thicknesses of 400 µm and 100 µm under different protocols (8s I, 8s II, 4s I, and 4s II). This approach was not intended to compare slice thicknesses in detail but to assess how sensitively each structure responded to changes in imaging modalities. Although 100 µm reconstructions yielded significantly higher scores overall than those with 400 µm, these results were not presented in detail, as this was not the primary focus of the study. Low to average scores, high SD and large MMD indicated enhanced sensitivity in detecting differences. Based on this analysis, eight representative structures were selected for further evaluation to avoid unnecessary analyses (see Results), specifically to address the main research question of comparing the imaging protocols.

In the second round, these eight structures were reassessed by three independent expert examiners (consultant neuroradiologist, ENT specialist, senior ENT surgeon) with extensive experience in middle ear imaging analysis across the various six fpVCT imaging protocols. Each examiner assigned a score to each structure once. Interobserver differences were not resolved by consensus, as the aim was not to analyse interrater variability but to generate a cumulative score. Therefore, the individual scores of the three examiners were summed for each structure and protocol to the “cumulative score”. The cumulative score thus represents the sum of the individual ratings (0–3 per structure) across the eight selected structures, resulting in a maximum achievable score of 24 per protocol. To ensure consistency and accuracy, all assessments were conducted under identical conditions. Image data, formatted as DICOM files, were loaded into the “Horos” Image Viewer. Examiners were blinded to skull specimen identification, slice thickness, protocol, and other imaging parameters. Evaluations adhered to DIN 6868–157 standards for image display systems. The sequence of images was randomized using “Research Randomizer” (Version 4.0, Urbaniak, G.C. & Plous, S., 2013) to minimize learning effects.

### Gray-scale profile analysis

Gray-scale profiles were generated using the open-source software “ImageJ” (imageJ.org). Image files, aligned to the same sectional planes using “3D-Slicer”^48^ (version 4.11.20210226), were imported into the program. The “region of interest” (ROI) was identified and marked using the selection tool, and the ROI configuration was saved in the “ROI Manager” for precise replication across different image files in the study series. The gray-scale profiles were then analyzed using the “Plot Profile” tool. Uniform y-axis scaling was applied to all profiles. To facilitate visual comparison, combinations of modalities (400 µm and 100 µm) were graphically represented using Adobe Photoshop (version 21.x, Adobe, San José, California, USA). The ROI markings were highlighted in yellow for clarity.

### Statistical analysis

Data analysis was conducted using SPSS statistical software (version 28, IBM, Armonk, New York, USA). A significance threshold of *p* ≤ 0.05 was applied. The influence of protocols on image quality was evaluated using the Friedman test, which assesses dependent samples without assuming a normal distribution for sample sizes larger than two. A post hoc pairwise comparison followed to identify significant differences between specific protocol pairs. To account for multiple testing, the significance level was adjusted using the Bonferroni-Holm correction.

Bivariate Pearson correlation analyses were conducted to examine relationships between image quality, examination time, and radiation dose. Normal distribution of data was verified using the Shapiro–Wilk test, with significant correlations assumed for *p* ≤ 0.05. Graphs were generated using GraphPad Prism (version 8.0, GraphPad Software, San Diego, USA).

## Supplementary Information

Below is the link to the electronic supplementary material.

Supplementary Material 1

## Data Availability

The datasets used and analyzed during the current study are available from the corresponding author on reasonable request.
