# Supplementary material for: Comparative evaluation of flat-panel volume CT protocols for high-resolution visualization of middle ear anatomy in human skull specimens
Source: Sci Rep. 2026 Jan 5;16:629. doi: 10.1038/s41598-025-33592-5 (PMC12775453; doi:10.1038/s41598-025-33592-5)
Supplement: Supplementary file 1 — Supplementary Material 1 [file 41598_2025_33592_MOESM1_ESM.pdf]

Supplementary Table 1: Descriptive statistics (mean score, standard deviation, and MMD; scoring scale 0-3; 0 = non definable – 3 = very good definability) calculated across **two slice thicknesses (400 µm vs. 100 µm) and four imaging protocols (8s I, 8s II, 4s I, and 4s II).**

	Mean score (0-3)	standard deviation	maximum-minimum difference
**Ligamentum mallei anterius**	1.75	0.77	3.00
**Ligamentum mallei superius**	1.44	0.73	3.00
**Ligamentum mallei laterale**	0.38	0.50	1.00
**Ligamentum incudis superius**	0.75	0.45	1.00
**Ligamentum incudis posterius**	1.50	0.89	3.00
**Musculus stapedius**	0.88	0.72	2.00
**Musculus tensor tympani in the Semicanal**	3.00	0.00	0.00
**Musculus tensor tympani (tympanal)**	2.00	0.63	2.00
**Articulatio incudomallearis**	1.56	0.73	2.00
**Articulatio incudostapedialis**	1.38	0.89	3.00
**Manubrium mallei**	3.00	0.00	0.00
**Processus lateralis mallei**	2.63	0.81	3.00
**Corpus incudis**	3.00	0.00	0.00
**Crus breve incudis**	2.94	0.25	1.00
**Crus longum incudis**	2.50	0.52	1.00
**Processus lenticularis**	2.06	0.85	3.00
**Caput stapedis**	1.38	0.62	2.00
**Crus anterius stapedis**	1.00	0.73	2.00
**Crus posterius stapedis**	1.13	0.72	2.00
**Basis stapedis**	2.81	0.54	2.00
**Prominentia canalis nervi facialis**	1.31	0.95	3.00
**Chorda tympani**	0.88	0.62	2.00
**Canalis nervi petrosi majoris**	1.50	0.89	3.00
**Fenestra ovalis**	3.00	0.00	0.00
**Fenestra rotunda**	3.00	0.00	0.00
**Membrana tympanica secundaria**	0.19	0.40	1.00
**Eminentia pyramidalis**	2.56	0.63	2.00
**Processus cochleariformis**	2.69	0.60	2.00

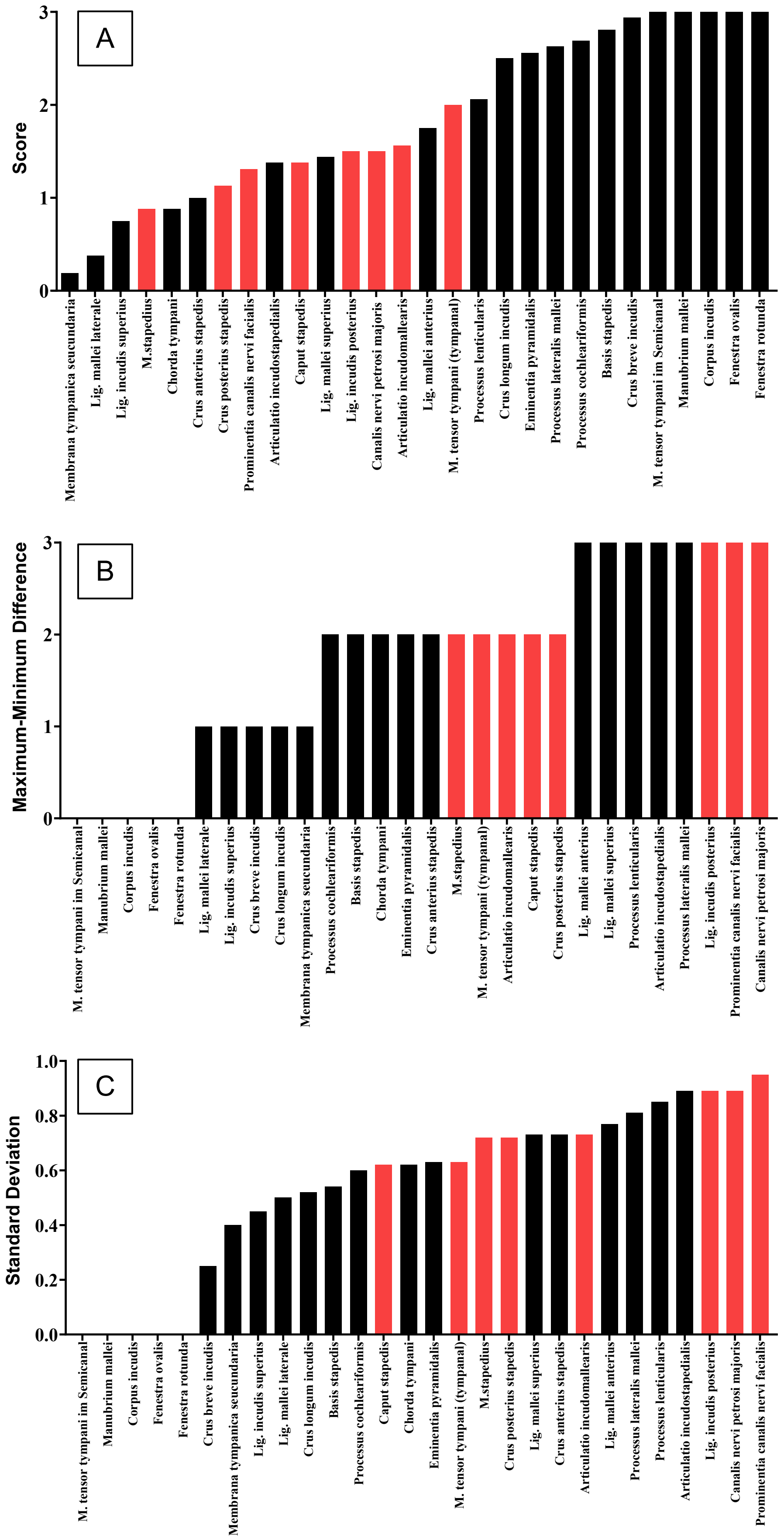

Supplementary Figure 1: Descriptive statistics of the scoring of 28 anatomical structures of the middle ear using fpVCT. *In each panel, the x-axis displays the structures sorted according to the parameter shown: (A) score, (B) maximum–minimum difference (MMD), and (C) standard deviation (SD). This ordering was chosen to identify structures with high discriminatory potential (low to average scores, large MMD and high SD). The structures selected for further evaluation in the middle ear region are highlighted in red. Scores were averaged over the slice thicknesses of 100 µm and 400 µm, as well as the protocols 8s I, 8s II, 4s I, and 4s II. Score: 0 = Not or not reliably definable; 1 = Poor definability; 2 = Good definability; 3 = Very good definability. Raters: n = 1. fpVCT = flat-panel volume CT.*
